# Integration of wastewater treatment into process design of lignocellulosic biorefineries for improved economic viability

**DOI:** 10.1186/s13068-020-1657-7

**Published:** 2020-02-03

**Authors:** Tyler Tobin, Rick Gustafson, Renata Bura, Heidi L. Gough

**Affiliations:** The School of Environmental and Forest Sciences, University of Washington, 4000 15th Avenue NE, Seattle, WA 98195-2100 USA

**Keywords:** Biofuel, Poplar, Wastewater treatment, Anaerobic treatment, Evaporator, Economic analysis, Ecosystem services, Industrial ecology, Ethanol

## Abstract

**Background:**

Production and use of bio-based products offer advantages over conventional petrochemicals, yet the relatively high cost of production has restricted their mainstream adoption. Optimization of wastewater treatment processes could reduce capital expenditures, lowering the barrier to market entry for lignocellulosic biorefineries. This paper characterizes wastewater associated with lignocellulosic ethanol production and evaluates potential wastewater treatment operations.

**Results:**

It is found that organic material is intrinsic to bioconversion wastewater, representing up to 260 kg of biological oxygen demand per tonne of feedstock processed. Inorganics in the wastewater largely originate from additions during pretreatment and pH adjustments, which increase the inorganic loading by 44 kg per tonne of feedstock processed. Adjusting the ethanol production process to decrease addition of inorganic material could reduce the demands and therefore cost of waste treatment. Various waste treatment technologies—including those that take advantage of ecosystem services provided by feedstock production—were compared in terms of capital and operating costs, as well as technical feasibility.

**Conclusions:**

It is concluded that wastewater treatment technologies should be better integrated with conversion process design and feedstock production. Efforts to recycle resources throughout the biofuel supply chain through application of ecosystem services provided by adjacent feedstock plantations and recovery of resources from the waste stream to reduce overall capital and operating costs of bioconversion facilities.

## Introduction

Bio-based products have potential to accelerate the sustainable development of the global economy. Bio-based products span a wide range of materials including liquid fuels, plastics, construction materials, adhesives and lubricants among others. Cultivation of biomass feedstocks stimulates rural economies [1]. Domestic cultivation and processing improves security of scarce resources [2]. Production and end use of bio-based products is typically less polluting in terms of both carbon dioxide emissions and other environmental impacts than conventional petroleum derived products [3–5].

Despite these benefits, bio-based products have not achieved mainstream adoption. Chief among the various obstacles holding back the bio-based economy is the inability of bio-based products to compete at the low price points of petrochemical alternatives [6]. The high cost of bio-based products stems from a combination of feedstock prices and extensive processing requirements, particularly for lignocellulosic feedstocks.

Biochemical conversion, or bioconversion, presents one promising platform to process biomass into a wide range of products. Bioconversion of lignocellulosic feedstocks incorporates four main unit operations: pretreatment to fractionate the biomass, hydrolysis to break down cellulose and hemicellulose polymers into carbohydrate monomers, fermentation to convert carbohydrates into desirable products, and separation to purify the desired products. The material stream remaining after separation is known as stillage which becomes the largest wastewater stream in bioconversion facilities [7]. Up to 20 L of stillage can be generated per liter of product [8].

Wastewater from lignocellulosic biorefineries is generally characterized by high-strength organic loading, but can vary based on feedstock and process implementation [9]. Stillage accounts for 85% of wastewater composition, other sources include flash condensate from steam explosion pretreatment processes, boiler and cooling water blowdown, and cleaning water [7].

Conventional starch to ethanol and spirits distilleries produce stillage wastewater similar in composition to lignocellulosic stillage [9, 10]. Various wastewater treatment methods have been explored for these wastes. One common treatment method is evaporation of the stillage into a syrup and subsequent spray drying of the syrup onto spent grains for production of animal feed known as dried distillers’ grains with solubles (DDGS) [11]. However, lignocellulosic processes do not produce spent grain and therefore are unable to take advantage of DDGS as a coproduct. Another common treatment method for high-strength organic wastewater is anaerobic treatment. Anaerobic treatment utilizes oxygen-free biological reactors to degrade organic material into a mixture of methane and carbon dioxide known as biogas which may be combusted as a natural gas substitute [12]. Biogas recovery, coupled with the lower energy demands of anaerobic treatment has made it the focus of lignocellulosic wastewater treatment research [7, 13]. However, the high capital cost of anaerobic reactors and the need for supplemental treatment escalates wastewater treatment to up to 21% of the total capital cost of a lignocellulosic biorefinery [7]. Less capital-intensive wastewater treatment processes will reduce the investment required for new facilities, thereby lowering the barrier to market entry for bio-based products.

This study has three primary objectives to better understand wastewater treatment in the context of lignocellulosic bioconversion: first, to determine how upstream processes impact the wastewater profile, second, to identify upstream process changes to minimize wastewater treatment requirements, and third, to screen wastewater treatment technologies which may reduce the capital investment required to construct a biorefinery. To achieve these objectives, a system-wide mass balance was generated from lab-scale experiments to determine how constituents move through the bioconversion process and during which processes wastes are generated. Then process models were used to assess the technical and economic influence of the proposed process alternatives.

## Methods

### Experimental methods

#### Raw material

Two-year-old 2nd cycle short-rotation coppice poplar used in this research is a hybrid of *Populus trichocarpa* and *Populus deltoides* (clone number 5077), obtained from a plantation near Jefferson, OR, managed by GreenWood Resources (Portland, OR). The poplar trees were harvested without leaves and chipped in fall 2015. Samples were stored at − 20 °C until processed.

#### Steam explosion

Steam explosion was conducted as previously described by Dou et al. [14]. In brief, 300 g oven-dried (OD) biomass was impregnated with 3% (w/w) sulfur dioxide (SO_2_) overnight, and then steam pretreated at 195 °C for 5 min in a 2.7-L batch reactor (Aurora Technical, Savona, BC, Canada). After steam explosion, the pretreated biomass slurry was separated into solid and liquid phases using vacuum filtration. The solid phase was then washed with deionized water to remove the free sugars.

#### Solid-phase saccharification and fermentation

Solid-phase saccharification and fermentation was performed to simulate commercial enzymatic hydrolysis and fermentation processes where enzymes would remain active through both hydrolysis and fermentation steps. Sterile flasks, media, sterile sampling technique were employed to maintain suitable environment for fermentation and to produce accurate, repeatable results.

##### Enzymatic hydrolysis

Enzymatic hydrolysis was carried out using cellulase (Celluclast 1.5 L, Sigma) at 20 filter paper units (FPU)/g cellulose and β-glucosidase (Novozyme 188, Sigma) at 40 cellobiase units (CBU)/g cellulose. The solid phase was hydrolyzed at 10% (w/v) water-insoluble content (WIS) in a total volume of 250 mL at 50 °C and 175 rotations per minute (rpm) in a shaker. 50 mM citrate buffer was added to maintain the pH at 4.8. After 48 h of enzymatic hydrolysis, the flask temperature was reduced to 30 °C and the pH increased to 6.0 using 1.0 M sodium hydroxide (NaOH) in preparation for fermentation as described in the following sections.

##### Yeast strain

*Scheffersomyces stipitis* ATCC 58376 (also-known-as: *Pichia stipitis* Y-7124) was obtained from ATCC, Manassas, Virginia.

The strain was taken from − 80 °C stocks and maintained on YPG solid medium (10 g/L yeast extract, 20 g/L peptone, 20 g/L glucose, and 18 g/L agar, Difco, Becton-Dickinson, MD) at 4 °C and transferred to fresh plates on a weekly basis.

##### Culture media conditions

Cells were grown to high cell density in foam-plugged 1-L Erlenmeyer flasks containing 500 mL liquid media with additional trace nutrients [10 g/L Macron Fine Chemicals Granular Glucose, 20 g/L Sigma-Aldrich d-(+)-Xylose (99%), 3 g/L BD Bacto Yeast Extract, 5 g/L BD Bacto Peptone, 2.3 g/L Fisher Chemical Urea, and 1 g/L Fisher Chemical magnesium sulfate heptahydrate (MgSO_4_ × 7-H_2_O)] in an orbital shaker for 48 h at 30 °C and 175 rpm, with a concurrent transfer to fresh medium performed every 24 h.

After 48 h of growth, cell culture suspension was centrifuged, and spent media decanted to yield cell pellets. Pellets were then washed three times with sterile distilled water and subsequently adjusted with sterile distilled water to form a concentrated yeast culture. The dry cell weight per liter (DCW/L) per liter of the concentrated yeast culture was measured on a spectrophotometer (Shimadzu UV-1700, Columbia, MD) via standard curves relating 600 nm absorbance to DCW/L concentration.

##### Fermentation

Yeast culture was added directly to the fermentation flasks without denaturing enzymes to allow for continued hydrolysis throughout the fermentation process. Concentrated yeast culture was added to achieve 5 g DCW/L media. Dry trace nutrients were added to supplement the fermentation media at following concentrations: 3 g/L yeast extract, 5 g/L peptone, 2.3 g/L urea, and 1 g/L MgSO_4_ × 7-H_2_O. Following addition of yeast, flasks were incubated at 30 °C and maintained with continuous agitation (175 rpm), and pH value of ~ 6.0.

#### Liquid-phase detoxification and fermentation

Early attempts to perform fermentation on untreated, steam exploded liquid phase were unsuccessful resulting in the need to detoxify the liquid phase prior to fermentation.

##### Detoxification

Powdered activated carbon (Fisher Scientific C272-500) was added to untreated, steam exploded liquid phase (pH = 1.6 ± 0.1) at a consistency of 10% (w/v) and agitated for 12 h at 175 rpm. Following treatment, the activated carbon was removed via vacuum filtration through a 0.2-µm sterile bottle filter. The pH was then adjusted to 6.0 using 50% (w/w) NaOH solution.

##### Fermentation

The same yeast strain, storage, cultivation, and harvest procedures as described above were employed for fermentation of detoxified liquid phase. Trace nutrients (3 g/L yeast extract, 5 g/L peptone, 2.3 g/L urea, and 1 g/L MgSO_4_ × 7-H_2_O) were added to the sterile, detoxified liquid phase to create the fermentation media. Concentrated yeast culture was added to achieve 5 g DCW/L media. Fermentation flasks were incubated at 30 °C and maintained with continuous agitation at 175 rpm.

#### Distillation

The resulting fermentation broths from solid phase and detoxified liquid-phase fermentation were distilled separately under the same conditions. Distillation was performed using an IKA RV 10 rotary evaporator and accompanying IKA HB 10 water bath (Staufen, Germany). Batches of fermentation broth were distilled 250 mL per batch to accommodate vessel size; 0.5 mL of anti-foam agent (Sigma Antifoam 204) was added to each batch. The rotary evaporator was set to rotate at 20 rpm and maintain a vacuum of 350 millibar. The water bath was maintained at a temperature of 87 °C. Distillation proceeded until visual signs of boiling ceased.

#### Compositional analysis

Several analytical methods were used to determine the composition of each process material stream.

##### Elemental analysis

Elemental analysis was conducted to quantitatively determine the inorganic constituents of biomass samples. The analysis was conducted by the University of Washington School of Environment and Forest Sciences Analytical Service Center. Solid biomass samples were ground to 40 mesh particle size and dried completely in a 105 °C oven. Oven dry samples were digested in accordance with Environmental Protection Agency (EPA) Method 3050B [15]. In brief, samples were mixed with concentrated nitric acid and refluxed at 95 °C ± 5 °C for 30 min then cooled and concentrated via evaporation. Following concentration, hydrogen peroxide was mixed with the sample digest until the sample was completely reacted, again the sample digest was concentrated via evaporation. Finally, concentrated hydrochloric acid was added to the sample digest and the resulting slurry was filtered. The sample digest filtrate was then analyzed on a Thermo Jarrell-Ash (Thermo Scientific) iCAP 61E Inductively Coupled Plasma Emission Spectrometer for Al, As, B, Ba, Ca, Cd, Cr, Cu, Fe, K, Mg, Mn, Mo, Na, Ni, P, Pb, S, Se, Zn, Si, and Ag.

##### Ash

Ash content of raw biomass samples was measured gravimetrically by heating 20-mesh-milled dry biomass to 575 °C for 12 h [16].

##### Solid fraction carbohydrates, acetate groups and acid-soluble lignin

The chemical composition of raw biomass and solid fraction was determined according to a modified method derived from TAPPI Standard Method T222 om-11 [17] and NREL protocols [18]. Briefly, 0.2 g of finely ground, oven-dried sample was treated with 3 mL 72% sulfuric acid (H_2_SO_4_) for 2 h at room temperature, then diluted into 120 mL total volume and autoclaved at 121 °C for 60 min. Klason lignin contents were determined by gravimetric methods by filtration through tared sintered glass crucibles. After filtration, the carbohydrate and acetyl composition of the filtrate was analyzed by HPLC (Dionex ICS-3000, as described in [19]) and the acid-soluble lignin (phenolics) in the filtrate was analyzed by UV spectrophotometer (Shimadzu, Tokyo, Japan) at 205 nm.

##### Liquid fraction carbohydrate, ethanol, and degradation products

The concentration of monomeric sugars was determined with a high-pressure liquid chromatography (HPLC) system (Dionex ICS-3000). The concentration of monomeric sugars, ethanol and degradation products, such as acetic acid, furfural and 5-hydroxymethylfurfural (5-HMF) were measured using refractive index detection on a Shimadzu Prominence LC, as described by Suko and Bura [19]. Monomeric and oligomeric soluble carbohydrates were determined using NREL LAP TP-510-42623 [18]. Phenolic concentration in the liquid fraction was assayed by the Folin–Ciocalteu method [20], using a ultra-violet (UV) spectrophotometer (Shimadzu, Tokyo, Japan) at 765 nm. Gallic acid was used as calibration standard.

##### Wastewater characteristics

Several wastewater-specific parameters were measured from the stillage streams to better characterize the wastewater stream. These parameters along with the equipment and methods used to perform the analysis are provided in Table 1.

**Table 1:** Wastewater characterization parameters and methods

Test	Reagents	Method
Total solids, total volatile solids, total suspended solids, volatile suspended solids	–	Standard Method 2540 [21]
Biological oxygen demand, 5 day	Hach BOD Nutrient Pillows, PolySeed Microbial Culture	Standard Method 5210 [21]
Alkalinity	–	Standard Method 2320B [21]
Chemical oxygen demand	Hach High Range TNT COD Digestion Vials (0–1500 mg/L)	Hach Method 8000 [22]
Reactive phosphorous	Hach High Range TNT Reactive Phosphorous (1.0–100 mg/L PO4)	Hach Method 8114 [22]
Total phosphorous	Hach High Range TNT Total Phosphorous (1.0–100 mg/L PO_4_)	Hach Method 10127 [22]
Ammonia	Hach High Range TNT Nitrogen-Ammonia (0.4–50 mg/L NH_3_-N)	Hach Method 10031 [22]
Nitrate	Hach High Range NitraVer 3 Nitrogen-Nitrate (0.2–30.0 mg/L NO_3_-N)	Hach Method 10020 [22]
Nitrite	Hach High Range NitraVer 3 Nitrogen-Nitrite (0.003–0.500 mg/L NO_2_-N)	Hach Method 10019 [22]
Total nitrogen	Hach High Range TNT Total Nitrogen (2.0–150 mg/L N)	Hach Method 10072 [22]
Sulfate	Hach High Range SufaVer 4 Reagent Pillows (0.0–70 mg/L SO_4_)	Hach Method 8051 [22]

### Economic modeling methods

Capital equipment costs were sourced from the literature or from personal communication with equipment vendors. All values were converted to 2016 United Stated Dollars (USD) using the Chemical Engineering Plant Cost Index [23].

Operating costs were calculated through aggregation of material and energy costs and fixed costs such as maintenance. Labor costs were not included in this analysis. Chemical prices were adjusted with the US Bureau of Labor Statistics Producer Price Index for Other Inorganic Chemicals [24]. Maintenance was assumed to cost 10% of equipment costs annually [25]. For comparison purposes energy flows were valorized as either electricity or steam. An electricity price of 0.06 USD/kWh is within the range of typical industry energy prices in the United States [26]. A steam price of 11.79 USD per 1000 kg of 62 bar, 455 °C steam was calculated using a natural gas boiler at 85% efficiency [27, 28].

All future cash flows were discounted a rate of 10% to incorporate the time-value of money [7].

This cost estimate attempts to incorporate all major equipment costs and known material and energy streams and may be considered accurate to within − 25% and + 30% of values presented [25].

## Results and discussion

### Bioconversion process mass flows

#### Carbohydrate and organic compound mass flows

**Fig. 1:**
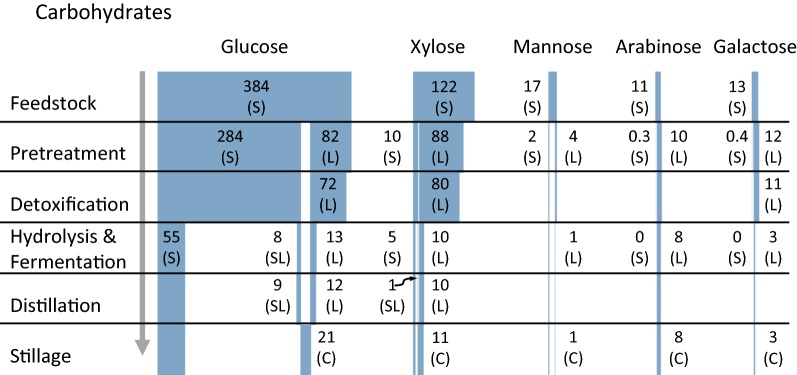
Carbohydrate material flows through the bioconversion process. Stillage material flows represent a contribution to wastewater. The figure should be read from top to bottom where shaded blocks represent the relative material flow of the named constituent at the end of each unit operation. Exact material flows are provided as numbers nearby the corresponding shaded block. Material flows represented as kg/tonne OD biomass. (S)—solid phase, (L)—liquid phase, (SL)—liquid phase derived from pretreated solid, (C)—combined stillage. All values are means of triplicates

**Fig. 2:**
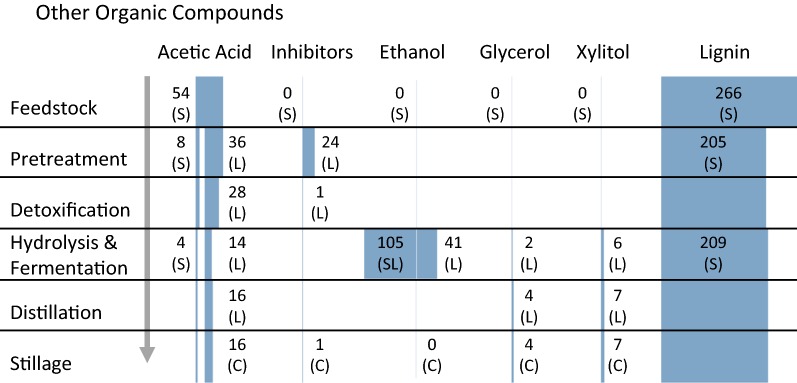
Other organic material flows through the bioconversion process. Stillage material flows represent a contribution to wastewater. The figure should be read from top to bottom where shaded blocks represent the relative material flow of the named constituent at the end of each unit operation. Exact material flows are provided as numbers nearby the corresponding shaded block. Material flows represented as kg/tonne OD biomass. (S)—solid phase, (L)—liquid phase, (SL)—liquid phase derived from pretreated solid, (C)—combined stillage. All values are means of triplicates

Figures 1 and 2 provide a summary of the mass flow rate of each measured organic compound throughout the bioconversion process. Pretreatment fractionated the raw biomass into solid and liquid phases. The solid phase accounted for 57.4 ± 1.31% of the raw biomass and was composed of predominately cellulose and acid-insoluble lignin. The liquid phase accounted for 42.6 ± 1.31% of the biomass and was composed of hydrolyzed hemicellulose (arabinose, galactose, glucose, xylose, mannose), acetic acid, sugar degradation products (furfural, 5-HMF), and lignin-derived phenolic compounds.

Early experiments indicated inhibitory compounds present in the liquid fraction limited the effectiveness of *P. stipitis* to ferment the liquid phase resulting in an ethanol yield of near 0% (w/w). Therefore, the liquid phase was detoxified with powdered activated carbon which resulted in 100% removal of furfural and HMF, 88% removal of total phenolic compounds, and 22% removal of acetic acid. Detoxification also resulted in a 11% (w/w) loss of the total carbohydrate content of the liquid phase.

The detoxified liquid phase was fermented with an ethanol yield of 25% (w/w) (gram ethanol per gram total carbohydrate) which equates to 48% of the theoretical yield. Overall, 83% of carbohydrates were consumed during liquid-phase fermentation. Of the remaining carbohydrates, 76% were carbohydrate oligomers and, therefore, inaccessible to the yeast during fermentation. The low ethanol yield is likely due to residual inhibitory compounds such as dibutyl phthalate, phthalic acid derivatives [29] and acetic acid which will lead to increased stress response mechanisms and reduced normal, ethanol producing metabolism [30, 31].

The solid phase was saccharified and fermented with an ethanol yield of 38% (w/w) (gram ethanol per gram total carbohydrate) which equates to 74% of the theoretical yield. Overall, 76% of carbohydrates were consumed during fermentation. Of the remaining carbohydrates, 98% were carbohydrate oligomers and, therefore, inaccessible to the yeast during fermentation.

Rotary evaporation provided 100% (w/w) ethanol removal for both liquid-phase and solid-phase fermentation broths. The concentration of carbohydrates in the liquid-phase and solid-phase fermentation stillage following distillation increased by a factor of 2.00, but carbohydrate mass flows remained nearly constant.

#### Inorganic compound mass flows

**Fig. 3:**
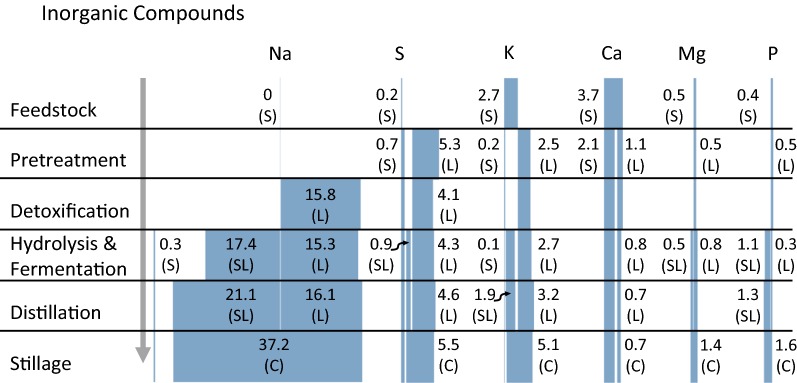
Inorganic material flows through the bioconversion process. Stillage material flows represent a contribution to wastewater. The figure should be read from top to bottom where shaded blocks represent the relative material flow of the named constituent at the end of each unit operation. Exact material flows are provided as numbers nearby the corresponding shaded block. Material flows represented as kg/tonne OD biomass, (S)—solid phase, (L)—liquid phase, (SL)—liquid phase derived from pretreated solid, (C)—combined stillage. All values are means of triplicates

Figure 3 provides a summary of the mass flow rate of the major inorganic compounds measured throughout the bioconversion process. Raw biomass is composed of 1.91 ± 0.04% ash content, measured gravimetrically. Of the ash fraction, calcium, potassium, magnesium, and phosphorous are the largest measured contributors at 47.5%, 35.0%, 6.5%, and 5.7%, respectively.

Following pretreatment, the inorganic constituent load increased by 72.7% due to the SO_2_ impregnation process with sulfur dominating the measured composition at 44.9% and calcium dropping to 24.1% of the total measured components for combined solid and liquid pretreated material. Similarly, pH adjustment (sodium hydroxide) and buffering solution (sodium citrate) additions, as part of the detoxification, fermentation, and saccharification steps, continued to increase the total inorganic loading. Following these steps, sodium became the most prevalent inorganic constituent accounting for 66.1% followed by sulfur at 12.0% including all solid and liquid material streams. The inorganic load remained unchanged following distillation, however, due to ethanol and water loss, inorganic concentrations increased by a factor of 2.00 in the combined stillage steam.

#### Wastewater characteristics and mass flows

Table 2 presents concentration and mass flow values for a range of wastewater characteristics. Data are presented for two cellulosic feedstocks, corn stover and poplar chips. Corn stover data were reproduced from the 2011 NREL Bioconversion Process report [32] and poplar data were measured over the course of this study.

**Table 2:** Characteristics of wastewater from corn stover-based bioconversion process reproduced from [32] and poplar-based bioconversion process (this study)

Parameter	Concentration (mg/L)		Process mass flow (normalized to kg/tonne OD biomass)	
	Corn stover	Poplar	Corn Stover	Poplar
BOD	33,000	32,100	135	160
Soluble BOD	32,600	32,300	130	160
Total COD	87,400	169,100	355	845
Soluble COD	84,600	78,700	340	390
Total solids	68,433	158,400	280	790
TVS	58,460	128,700	240	640
TSS	1500	73,100	6.10	365
VSS	1360	68,500	5.5	342
TDS	66,933	85,200	270	425
Ammonia-N	1060	160	4.3	0.8
Total P	805	2030	3.3	10.1
Ortho-P	805	1275	3.3	6.4
Total alkalinity	2750	3210	11.2	16.0
Sulfate	4400	1820	17.9	9.1
Silica	1580	10	6.4	0.1
Ba	0.015	0.927	< 0.01	< 0.01
Cd	<0.001	< 0.046	< 0.01	< 0.01
Ca^2^	6.790	146	0.03	0.73
Cr	0.177	2.33*	< 0.01	0.01
Cu	0.005	< 0.019	< 0.01	< 0.01
Fe	0.814	11.9	< 0.01	0.06
Pb	0.003	< 0.070	< 0.01	< 0.01
Mg	4.630	298	0.02	1.49
Mn	0.096	0.373	< 0.01	< 0.01
K	498	1020	2.03	5.10
Cl	2473	NA	10.06	NA
Na	15.8	7450	0.06	37.17
St	0.086	NA	< 0.01	NA

*NA* not analyzed, *BOD* biochemical oxygen demand, *COD* chemical oxygen demand, *TVS* total volatile solids, *TSS* total suspended solids, *VSS* volatile suspended solids, *TDS* total dissolved solids

* Denotes exceedance of monthly discharge limitations for organic chemical facilities (40 CFR §414.91 2017)

Most parameters show similar trends in the composition of wastewater produced from corn stover and poplar. However, large differences can be observed for several parameters including total chemical oxygen demand (COD), total solids and total volatile solids which can be explained through methodology differences between the two studies. In the corn stover study (NREL), all parameters were measured following insoluble lignin separation, but in the poplar study (this study) total COD, total solids and total volatile solids parameters were measured while insoluble lignin was still present in the wastewater stream, greatly increasing the values in the poplar wastewater.

Other discrepancies, including phosphorous (ortho-P and total P), ammonia, sodium and silica, are likely the result of processing differences between the two studies. The total phosphorous content of corn stover ranges from 0.65 to 0.88 g/kg [33], while the total phosphorous content of unprocessed poplar chips was measured at 0.4 g/kg (Fig. 3). Also shown in Fig. 3, is a sharp increase in the phosphorous content following fermentation, this suggests that nutrient additions during fermentation drive the phosphorous loading of the wastewater stream. In the corn stover study, pH was adjusted with ammonia following pretreatment, while sodium hydroxide was used in the poplar study resulting in large differences between ammonia and sodium values observed in Table 2. Finally, the harvest method of corn stover (bailing) results in greater surficial soil and, therefore, silica on the feedstock as compared to chipped poplar.

### Impacts of upstream processes on wastewater profile and alternatives

#### Carbohydrate and organic compound impacts and alternatives

Residual carbohydrates in the combined stillage stream amount to 44 kg/OD tonne feedstock as shown as the sum of all carbohydrates in the combined stillage phase in Fig. 1. Carbohydrates in the stillage phase represent wasted resources impacting the overall process yield. Improved processing techniques at the commercial scale including mechanical mixing during solid-phase hydrolysis [34, 35], acclimated yeast strains [36, 37], and combined solid- and liquid-phase fermentation [7] could reduce the quantity of carbohydrates in the stillage stream. Regardless of carbohydrate recovery, organic matter is the largest fraction of wastewater constituents and should be primary focus of treatment alternatives.

#### Inorganic compound impacts and alternatives

Most of the wastewater inorganic load is composed of constituents added during processing (Fig. 3), therefore, process engineers have a high degree of control over the inorganic composition of the wastewater.

Pretreatment is one area where inorganic loading may be controlled. SO_2_ impregnation results in the addition of over 27 times the original sulfur content of the biomass. The sulfur content of wastewater streams is important, particularly when anaerobic digestion is part of the treatment process. Sulfate is readily reduced to hydrogen sulfide (H_2_S) during anaerobic digestion and may compose up to 1.3% (w/w) of biogas produced from lignocellulosic stillage [7]. Due to its corrosive nature, it is not recommended to combust fuels containing over 1% (w/w) H_2_S [38]. In addition, biogas with H_2_S concentration higher than 0.004% (w/w) is not recommended for integration into natural gas pipelines [38]. Therefore, H_2_S treatment is necessary for any practical application of biogas given current sulfate concentrations.

H_2_S treatment can be costly. For example, in an NREL biorefinery design study, purchase of lime to operate flue gas scrubbers which entrain sulfur emissions as gypsum (CaSO_4_) amounts to $2.2 million per year or about 2% of all operating expenditures [7]. Pretreatment is the only unit operation which requires the addition of sulfur compounds and biomass has a relatively low sulfur content; removal of the sulfur catalyst from the steam explosion step would withdraw the need for flue gas desulfurization. Substitution of SO_2_ with a different acid such as nitric or phosphoric acid is one potential solution [39]. Additionally, many other sulfur-free pretreatment technologies have been developed: fungal pretreatment, mechanical comminution, organosolv, ozonolysis, ionic liquids, liquid hot water, ammonia fiber explosion (AFEX), wet oxidation, and CO_2_ explosion, among others [40]. Further development of these technologies and corresponding economic analyses may identify a pretreatment method with overall lower costs than acid-catalyzed steam explosion.

pH adjustment is another unit operation which adds to the inorganic wastewater load. pH adjustment occurs following pretreatment to condition the liquid phase and to buffer pH throughout solid-phase saccharification and fermentation. In this study, sodium hydroxide was used to adjust pH and sodium was observed as the most prevalent inorganic constituent in the combined stillage stream. This correlation shows that pH adjustment is a primary driver behind which ions will compose the inorganic fraction of the wastewater. Given this large impact, care should be taken to determine which chemicals are used to adjust pH. Table 3 presents a comparison of several chemicals with respect to chemical cost, neutralization salt parameters, and downstream treatment methods.

**Table 3:** A comparison of the cost, neutralization salt characteristics and treatment methods of probable neutralizing agents for use during the bioconversion process

Neutralizing agent	Chemical cost (USD/tonne OD feedstock)	Primary salt and parameters	Downstream treatment methods
Ammonium hydroxide (NH_4_OH)	18.50 [7]	(NH_4_)_2_SO_4_ Soluble Biological nutrient [41]	Biological nitrogen removal Ion exchange Reverse osmosis Electrodialysis
Sodium hydroxide (NaOH)	14.50 [42]	Na_2_SO_4_ Soluble Inhibitory to anaerobic processes [12]	Ion exchange Reverse osmosis Electrodialysis
Calcium hydroxide (Ca(OH)_2_)	6.90 [7]	CaSO_4_ Insoluble Carbohydrate loss [7]	Precipitation Ion exchange Reverse osmosis Electrodialysis
Potassium hydroxide (KOH)	153.50 [42]	K_2_SO_4_ Soluble Biological nutrient [43] Inhibitory to anaerobic processes [12]	Ion exchange Reverse osmosis Electrodialysis
Calcium carbonate (CaCO_3_)	28.60 [42]	CaSO_4_ Insoluble Carbohydrate loss [7]	Precipitation Ion exchange Reverse osmosis Electrodialysis

The ideal neutralizing agent would be of low cost, non-inhibitory to downstream biological processes and easily treated from wastewater streams. Ammonium hydroxide and sodium hydroxide most nearly meet these requirements. Chemical cost was calculated through stoichiometric substitution of each chemical for ammonium hydroxide in the 2011 NREL Aspen Plus model [7]

The salts formed during neutralization are an important operational consideration. This analysis assumes sulfate as the primary anion in solution following SO_2_-catalyzed steam explosion pretreatment resulting in generation of sulfate salts during neutralization. Insoluble salts cause additional wear and tear on equipment and are typically removed to limit equipment damage and scaling issues. Removal of insoluble salts requires two distinct solid–liquid separation operations. In the first, the pretreated slurry must be separated into solid and liquids fractions to prevent precipitated salts from becoming entrained in the pretreated solid and then, in the second, precipitated salts are separated from the conditioned liquid phase. Precipitation of salts has been shown to cause carbohydrate losses of up to 13% affecting overall process yield [7]. Soluble salts, on the other hand, present downstream treatment challenges. Monovalent ions have been shown to cause inhibitory effects on methanogens, an essential microbial community in anaerobic treatment systems, at concentrations as low as 3500 mg/L [12]. Soluble salts often require high energy separation techniques such as reverse osmosis or ion exchange processes [12]. However, some neutralization salts, such as ammonium sulfate, can be beneficial by providing a source for essential nutrients (e.g., N, K, S) which may compliment or offset other nutrient additions necessary for fermentation.

Given the relatively few options available for pH adjustment and their respective advantages and disadvantages it appears ammonium hydroxide or sodium hydroxide would provide the most compatible, treatable, and economical alternatives. If biological wastewater treatment is employed ammonium hydroxide may be the better alternative for its ease of treatment and nutritional benefits to the fermentation and wastewater treatment processes. If physical wastewater treatment is employed sodium hydroxide may be the better alternative given its lower cost. Alternatively, a combination of both ammonium hydroxide and sodium hydroxide may be used in tandem to limit the accumulation of any one cation in the wastewater stream. Use of mixed ammonium hydroxide and sodium hydroxide also allows for control over nitrogen loading of the wastewater which is an important parameter to consider during biological wastewater treatment. Often, nitrogen must be added to during treatment as a necessary nutrient for microbial communities. Use of enough ammonia hydroxide to provide nitrogen for wastewater treatment and supplementing with sodium hydroxide to achieve the desired pH adjustment could result more treatable wastewater and potential cost savings.

#### Wastewater characteristics impacts and alternatives

The many similarities in the wastewater profile produced from corn stover and poplar chips suggest that feedstock choice is not a major driver of wastewater composition, though some consideration should be given to silica content to reduce wear on equipment. Generally, biorefinery wastewater will possess high organic loading, residual alkalinity and a mixture of inorganic compounds. However, processing alternatives do seem to have an impact on wastewater composition, particularly of the composition of inorganic compounds. As discussed in the previous section and seen again in Table 2, chemicals used for pH adjustment are a strong driver of the inorganic composition of biorefinery wastewater.

### Wastewater treatment design

Design of wastewater treatment systems specifically for lignocellulosic biorefineries is an emerging area of study. Among the few comprehensive wastewater treatment system designs for lignocellulosic biorefineries is the treatment system proposed in the 2011 NREL study which centers on anaerobic treatment [7, 32]. The NREL treatment system was designed to provide robust treatment of the wastewater stream allowing for direct re-use of treated water in upstream processes, however, the design accounts for approximately 21% of the capital cost of the biorefinery. Evaluation of less costly process alternatives may help reduce the overall capital cost of wastewater treatment systems and lower a barrier to market entry.

The design basis for this study is a 2000 tonne per day poplar to ethanol facility which generates a wastewater stream at a rate of 340 m^3^/h. Prior to wastewater treatment, solids will be separated from the stillage stream with a filter press and used as combustible material for the boiler. Therefore, the wastewater stream is assumed to have a similar composition to that of the soluble components of the poplar bioconversion wastewater stream described in Table 2 plus an additional 1 g/L of insoluble solids passing the filter press.

#### Treatment technology overview and alternatives

##### Anaerobic treatment (base case)

**Fig. 4:**
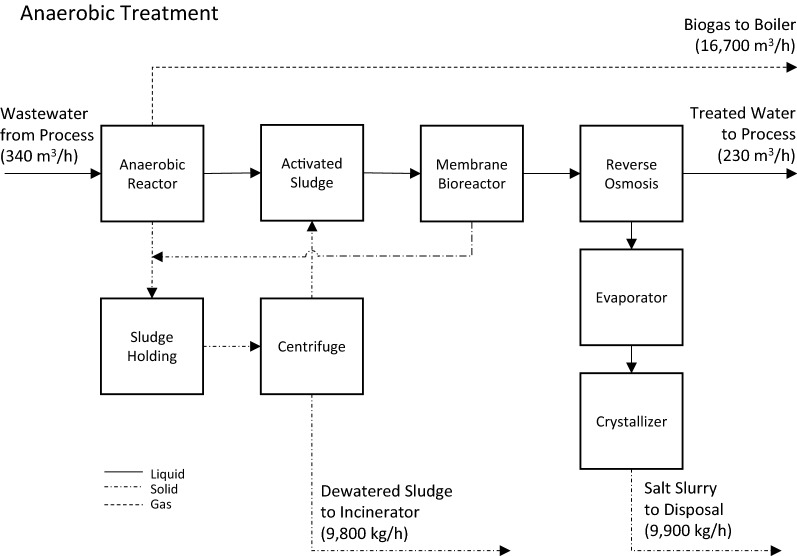
Anaerobic treatment process flow diagram

The anaerobic treatment systems proposed by NREL was selected as the base case from which to evaluate process alternatives. A process flow diagram of the anaerobic treatment system is presented in Fig. 4 as specified by NREL [7, 32]. In brief, an anaerobic reactor converts 91% of organic waste into biogas and cell mass. Activated sludge reactors are used to convert most of the remaining organic waste into carbon dioxide and cell mass while simultaneously converting ammonium to nitrate. A membrane bioreactor separates the activated sludge from the partially treated wastewater which proceeds to a reverse osmosis (RO) system for final treatment of salts and residual organic waste. Waste sludge is dewatered with a press and incinerated in the boiler. RO reject is evaporated and crystallized and disposed of off-site. The treated water is recycled back into the bioconversion process.

##### Ecosystem services

Ecosystem services are broadly defined as benefits freely gained through the function of natural environments. In the context of wastewater treatment, natural processes have a large capacity to degrade and filter impurities when well managed. In the NREL treatment system, anaerobic and aerobic reactors provide removal of 99.6% of soluble COD. The RO system, and corresponding treatment chain (evaporator and crystallizer), are used as a final polishing step to filter the residual organic matter and dissolved salts prior to water reuse. Substitution of ecosystem services for the RO treatment chain could reduce capital and operating cost of the overall treatment system.

Following processing in the membrane bioreactor, partially treated effluent will be discharged to an adjacent poplar plantation for use as reclaimed irrigation water. The EPA suggests reclaimed water be treated to at least 30 mg/L BOD and 30 mg/L TSS [44]. The membrane bioreactor effluent is designed provide treatment to approximately 280 mg/L COD [32]. However, dilution with fresh irrigation water will reduce discharge COD to recommended levels. During periods when irrigation is not required, partially treated wastewater may be stored in holding ponds. In regions where irrigation is not required, less restrictive permits may be granted for discharge of reclaimed water with higher concentrations of BOD and TSS [44]. By way of example, a bioconversion facility which processes 2000 tonnes per day would require approximately 33,000 hectares of plantation assuming poplar productivity of 22 OD tonnes per hectare per year (Greenwood Resources personal communication). Utilizing reclaimed water for irrigation distributed over the plantation would provide about 0.01 hectare-meters of irrigation, or about 3.2% of the average irrigation rate at the Greenwood Resources poplar test plot in Clarksburg, CA (Greenwood Resources personal communication). Therefore, using fresh irrigation water as make up water, the final COD discharge concentration would be approximately 30 mg/L.

Poplar trees are robust plants with tolerance to harsh conditions. Poplars are adept at capturing and absorbing nutrients (N and P) from the soil and have been used for phytoremediation purposes to reduce nutrient run-off [45, 46]. Therefore, it is recommended to use reagents amenable to uptake by poplar trees in upstream processes (e.g., ammonium hydroxide for pH adjustment, see “Impacts of upstream processes on wastewater profile and alternatives” section) to fully take advantage of ecosystem services. Poplars have also been shown to have little growth impairment up to total dissolved solids (TDS) content of 6000 mg/L in irrigation water and remain tolerant to TDS content up to 12,400 mg/L [47, 48]. Since no direct treatment of TDS is provided in this treatment scheme most inorganic constituents are expected to pass through the system yielding a predicted TDS content of up to 10,400 mg/L. However, dilution would, again reduce concentration of TDS reaching the plantation to approximately 1100 mg/L which is below the EPA recommended salinity for agricultural reclaimed water use of less than 2000 mg/L for non-sensitive crops [44].

Using ecosystem services provided by an adjacent poplar plantation for final wastewater treatment polishing would replace the need for a RO treatment chain. This would reduce capital costs by the $2.2 million or 4.4% of the total capital cost of the treatment system. Operation costs could be reduced by $113,000 annually or 3.6%.

##### Physical treatment (evaporation)

Biological treatment is a proven, reliable method for treating wastewater. However, the highly concentrated wastewater observed at biorefineries is in the upper range of COD concentrations for which anaerobic digestions systems are designed [12]. Biological systems operating near the edge of their design envelop are subject to stability issues and can be sensitive to shock loads or changing conditions. Physical treatment systems, such as evaporation, do not rely upon microbial communities and therefore can be more stable when properly maintained. As an alternative to biological treatment methods, evaporation has been evaluated to assess its feasibility as a treatment method at a lignocellulosic biorefinery.

Evaporation is commonly used in corn ethanol facilities where thin stillage is concentrated into a syrup called condensed distillers’ solubles (CDS) [11]. CDS is then combined with wet distillers’ grains and dried to form dried distillers’ grains with solubles (DDGS) which is sold as animal feed [11]. Multiple effect evaporators are commonly used in kraft pulp mills to concentrate spent liquors prior to combustion in a recovery furnace. Part of the motivation for evaporation and combustion of the organic solids is to significantly reduce wastewater treatment requirement of the mill. Evaporation has been evaluated for lignocellulosic ethanol production but has not gained much traction due to high energy costs and limited availability of boilers suited for high ash combustion [7, 49].

Evaporators come in many different varieties and configurations. Multi-effect systems arrange several evaporator units in series using the latent heat of the vapor from the previous unit to drive the next unit resulting in far greater efficiencies than single-effect systems [50]. The steam economy (kg vapor evaporated/kg steam feed) is increased roughly proportionally to the number of effects in the system, however the benefit of greater economy is balanced by increased capital cost for each effect.

**Fig. 5:**
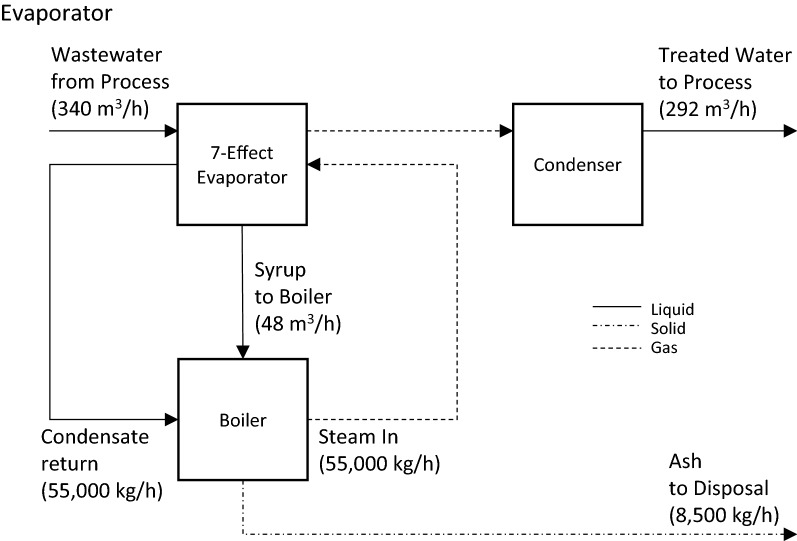
Evaporation treatment process flow diagram

A process flow diagram of the wastewater evaporation system is provided in Fig. 5. Most of the suspended solids (insoluble lignin) will have been removed from the wastewater stream leaving behind a thin stillage with approximately 8.5% dissolved solids; roughly 70% of dissolved solids are organic and 30% are inorganic.

The stillage is concentrated from 8.5% dissolved solids to approximately 60% solids with a seven-effect evaporation system. The system was modeled in WinGEMS software to determine the evaporator surface area and steam requirements [51]. Table 4 presents a list of inputs and outputs from the WinGEMS simulation. The steam requirement to run the evaporators is 55,200 kg/h which accounts for 23.5% of the steam production of the boiler currently specified by the NREL model [7]. Use of this steam for evaporation purposes would still allow the boiler and turbo-generator to meet all process steam and electricity demands of the biorefinery but would reduce the quantity of excess power exported to the grid from 13 to 8.3 MW.

**Table 4:** Evaporator design parameters obtained from a WinGEMS simulation of a seven-effect evaporator using initial conditions of measured wastewater parameters

Parameter	Unit	Value
*Input*		
Influent flow rate, *Q*	L/h	340,000
Influent temperature, *Q*_temp_	°C	80
Influent fraction solids, *X*_in_	–	0.085
Steam pressure, *S*_press_	bar	4.4
Steam temperature, *S*_temp_	°C	115
*Output*		
Steam demand, *S*_in_	kg/h	55,200
Evaporator area, SA	m^2^	2500
Effluent fraction solids, *X*_out_	–	0.61
Effluent pressure, *P*_out_	bar	0.28
Capacity, *C*	kg/h	295,000
Economy, *E*	kg/kg	5.34
Syrup flow rate, *Q*_syrup_	L/h	47,700
Condensate flow rate, *Q*_out_	L/h	292,000

The 60% solids syrup produced from the evaporators will be combusted in the furnace. Assuming the organic solids of the syrup have a heating value similar to dried sewage sludge (12.56 MJ/kg) then the 60% solids syrup will have an estimated lower heating value of 4.43 MJ/kg [52]. Ash from the furnace will be disposed of at an off-site landfill.

Vapor from the evaporation process will be condensed and recycled into the bioconversion process. The condensed liquid will contain organic compounds volatilized during the evaporation process. Studies have shown that use of stillage derived condensates for process water have little to no impact on fermentation yields [53, 54]. Therefore, the condensates will receive no further treatment prior to integration with bioconversion process water.

#### Economic analysis

A summary of the equipment, installed, and operation costs for each treatment alternative is presented in Table 5 and a breakdown of operating costs is presented in Table 6. Energy is the major driver of operation cost for all three treatment alternatives.

**Table 5:** Summary of equipment, installed and operating cost for treatment alternatives

Treatment alternative	Equipment cost ($)	Installed cost ($)	Operating cost ($/year)	Total cost, 30-year net present value ($)
Anaerobic treatment (base case)	27,200,000^a^	50,000,000 [7, 32]	3,182,000	80,076,000
Ecosystem services	26,023,000^a^	47,816,000 [7, 32]	3,069,000	76,747,000
Physical treatment (evaporation)	15,000,000^b^	33,000,000^b,c^	2,353,000	55,184,520

^a^Anaerobic treatment equipment cost estimated

^b^Personal communication with Lundberg, LLC | A Dustex Company

^c^Evaporator installed cost estimated at 2.2 times equipment cost as described by economic methods of [7]

**Table 6:** Operation cost comparison of wastewater treatment alternatives

Unit operation	Material/energy flows	Units	Quantity	Cost per Unit	Cost per h	Annual cost	Notes and sources
Anaerobic treatment						3,182,000	
Anaerobic reactor	Biogas (out)	kg/h	− 86,000	0.0118	− 1015	− 8,534,500	Credited as steam/[7, 27, 28]
	Heat (in)	kg/h	265	0.0118	3	26,300	Billed as steam/[7, 27, 28]
	Caustic (in)	kg/h	2240	0.2217	497	4,176,500	[7]
Aerobic reactor	Aeration electricity (in)	kWh/h	4280	0.06	257	2,159,700	[7, 26]
Sludge handling	Electricity (in)	kWh/h	1	0.06	0	500	[7, 26]
	Sludge (out)	kg/h	9760		0	0	Not accounted
Reverse osmosis	Electriticy (in)	kWh/h	50	0.06	3	25,200	[7, 26]
Evaporator	Heat (in)	kg/h	1010	0.0118	12	100,200	Billed as steam/[7, 27, 28]
Crystallizer	Salts (out)	kg/h	9870	0	0	0	Not accounted
Other	Other electrical	kWh/h	3050	0.06	183	1,539,000	[7, 26]
	Maintenance	–	–	–	–	3,688,800	10% of equipment capital
Ecosystem services						3,069,000	
Anaerobic reactor	Biogas (out)	kg/h	− 86,000	0.0118	− 1015	− 8,534,500	Credited as steam/[7, 27, 28]
	Heat (in)	kg/h	265	0.0118	3	26,300	Billed as steam/[7, 27, 28]
	Caustic (in)	kg/h	2240	0.2217	497	4,176,500	[7]
Aerobic reactor	Aeration electricity (in)	kWh/h	4280	0.06	257	2,159,700	[7, 26]
Sludge handling	Electricity (in)	kWh/h	1	0.06	0	500	[7, 26]
	Sludge (out)	kg/h	9760		0	0	Not accounted
Other	Other electrical	kWh/h	3050	0.06	183	1,539,000	[7, 26]
	Maintenance	–	–	–	–	3,688,800	10% of equipment capital
Physical treatment						2,353,000	
Evaporator	Steam (in)	kg/h	55,225	0.0118	652	5,480,400	Billed as steam/[27, 28]
	Syrup (out)	kg/h	− 62,140	0.0118	− 733	− 6,166,700	Credited as steam/[27, 28]
	Ash (out)	kg/h	1200	0	0	0	Not accounted
Other	Other electrical	kWh/h	3050	0.06	183	1,539,000	[26]
	Maintenance	–	–	–	–	1,500,000	10% of equipment capital

All costs are in USD. Annual costs assume 8410 h of operation

**Fig. 6:**
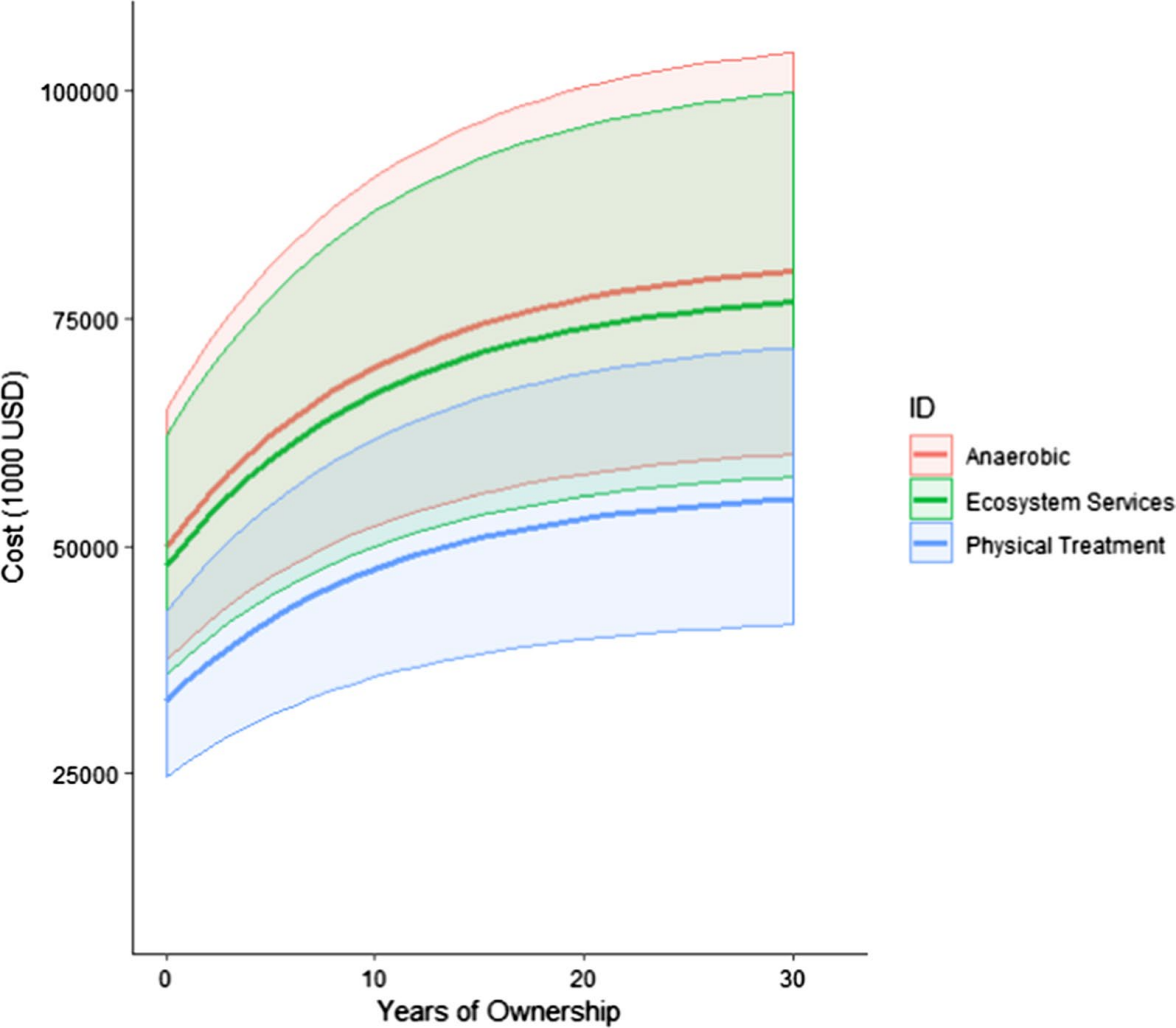
Wastewater treatment alternative cost of ownership. Bold line indicates predicted cost of ownership. Shaded area indicates estimation uncertainty (− 25%, + 30% of predicted). Cost of ownership calculated as net present value of installed cost plus operational costs discounted at 10% IRR

With the lowest capital and operating costs, physical treatment presents the most attractive economic scenario, followed by ecosystem services. Despite these predictions, cost estimation is an inherently uncertain procedure. Figure 6 shows the present worth of each treatment alternative over the 30-year facility lifetime, uncertainly is represented by the shaded region. Overlap in the uncertainty between all three treatment alternatives suggests that more accurate cost estimates are necessary to gain a better understanding of the true cost of each treatment alternative.

## Conclusion

Wastewater management will play an important role in the commercial development of lignocellulosic biorefineries. Analysis of bioconversion material flows shows that the composition of the wastewater stream can be manipulated through process adjustments. Efforts to reduce inorganic additions to the wastewater stream during pretreatment and pH adjusting processes may significantly reduce treatment demands and lower treatment cost.

Alternative treatment methods may also offer solutions to lower treatment costs. Taking advantage of ecosystem services which utilize feedstock plantations for tertiary treatment may help externalize treatment costs away from engineered systems into natural systems, while simultaneously improving crop yield. Evaporation of wastewater offers an operationally attractive means to treating wastewater which incorporates various resource recovery options, however, capital costs remain significant.

Most importantly, it has been demonstrated that wastewater treatment plays an integral role in the operations of a biorefinery. Design decisions of upstream processes impact the composition of the wastewater streams which in turn dictate the needs for down stream treatment. Similarly, resource recovery during wastewater treatment can reduce the need for virgin materials such as natural gas and fresh water. Processes designs should attempt to minimize wastewater treatment needs while maximizing recovery of valuable resources during treatment. To do so, upstream processes and wastewater treatment should be designed as an integrated system instead of as distinct processes.

## Data Availability

The datasets used and/or analyzed during the current study are available from the corresponding author on reasonable request.

## Abbreviations

5-HMF: 5-hydroxymethylfurfural
AFEX: ammonia fiber explosion
Ba: barium
BOD: biological oxygen demand
Ca: calcium
CaSO_4_: calcium sulfate, gypsum
CBU: cellobiase units
Cd: cadmium
CDS: condensed distillers’ solubles
Cl: chlorine
CO_2_: carbon dioxide
COD: chemical oxygen demand
Cr: chromium
Cu: copper
DCW/L: dry cell weight per liter
DDGS: dried distillers’ grains with solubles
EPA: Environmental Protection Agency
Fe: iron
FPU: filter paper units
g: gram
g/kg: gram per kilogram
g/L: grams per liter
h: hours
H_2_S: hydrogen sulfide
H_2_SO_4_: sulfuric acid
HPLC: high-pressure liquid chromatography
K: potassium
K_2_SO_4_: potassium sulfate
kg/h: kilograms per hour
L: liter
L/h: liters per hour
M: molar
m^3^/h: cubic meters per hour
Mg: magnesium
mg/L: milligrams per liter
MgSO_4_ × 7-H_2_O: magnesium sulfate heptahydrate
MJ/kg: megajoule per kilogram
mL: milliliters
mM: millimolar
N: nitrogen
NA: not analyzed
Na: sodium
Na_2_SO_4_: sodium sulfate
NaOH: sodium hydroxide
(NH_4_)_2_SO_4_: ammonium sulfate
nm: nanometers
NREL: National Renewable Energy Laboratory
OD: oven-dried
P: phosphorous
Pb: lead
RO: reverse osmosis
rpm: rotations per minute
S: sulfur
SO_2_: sulfur dioxide
St: strontium
TDS: total dissolved solids
TSS: total suspended solids
TVS: total volatile solids
USD: United States Dollars
USD/kWh: United States Dollars per kilowatt hour
UV: ultra-violet
VSS: volatile suspended solids
w/v: weight per volume
w/w: weight per weight
WIS: water-insoluble content
